# Exploring the professional and personal impact of migraine: a cross-sectional study in Greece

**DOI:** 10.22514/jofph.2025.031

**Published:** 2025-06-12

**Authors:** Maria Axiotidou, Theodoros Karapanayiotides, Doxa Papakonstantinou

**Affiliations:** ^1^Department of Educational and Social Policy, University of Macedonia, 54636 Thessaloniki, Greece; ^2^School of Medicine, Aristotle University of Thessaloniki, 54636 Thessaloniki, Greece

**Keywords:** Migraine, MIDAS, Disability, HEADWORK, Work difficulties, Productivity

## Abstract

**Background**: Migraine is a chronic neurological disorder affecting over one billion individuals globally. It is a leading cause of disability, significantly impacting daily functioning, social relationships and work-related productivity. This study aims to explore the impact of migraine-related disability in everyday life in terms of missed days and productivity loss, as well as to identify work-related difficulties associated with migraine and the potential factors that exacerbate these difficulties. **Methods**: This cross-sectional study was conducted from October 2023 to June 2024, involving 604 adult patients with migraine in Greece, selected through a convenience sampling method. Data collected included socio-demographic and clinical information, obtained using two validated self-reported questionnaires: the Migraine Disability Assessment (MIDAS) and the HEADWORK questionnaire. Statistical analysis was performed using IBM SPSS (Version 20.0), and descriptive statistics, chi-square tests, *t*-tests and Spearman’s correlation were employed to evaluate the relationship between disability and work-related challenges. **Results**: The MIDAS score revealed a high level of disability, with 52.2% of participants classified in the Severe Disability grade. HEADWORK scores highlighted moderate-to-severe work-related difficulties, particularly concerning stress management and environmental factors such as noise and brightness. Females, patients experiencing migraine with aura, and individuals with chronic migraine exhibited higher MIDAS and HEADWORK scores. **Conclusions**: This study reveals the substantial impact of migraine on professional productivity and social relationships, emphasizing challenges in work-related performance and daily activities. The findings underscore the need for workplace accommodations and targeted interventions to improve the quality of life of individuals with migraine.

## 1. Introduction

Migraine is a chronic neurological disorder characterized by recurrent headache episodes associated with symptoms such as nausea, vomiting, sensitivity to light (photophobia), sensitivity to sound (phonophobia) and visual disturbances [1]. Migraine is classified as either episodic (<15 headache days/month averaged over the last three months) or chronic (≥15 headache days/month averaged over the previous three months) based on the frequency of headache days [2]. The severity of migraine is most commonly evaluated based on the intensity and frequency of the headache [3]. Migraine is further divided into two subtypes: with or without aura [4].

More than one billion individuals are estimated to suffer from this primary headache disorder globally [5], with a prevalence of 15% [6]. Migraine is a leading cause of disability globally [7]. More importantly, migraine is the second highest specific cause of disability worldwide and remains the top cause of disability-adjusted life years (DALYs) in young women, as indicated by the Global Burden of Disease Study 2016 [8, 9]. Remarkably, no other disease results in as many years of healthy life lost in this age group, despite migraine not being associated with mortality [8]. Moreover, despite its debilitating effects, migraine remains underdiagnosed and undertreated [7].

Migraine can affect everyday life, including daily functioning and social aspects such as family and personal relationships [9]. Its prevalence tends to increase between the ages of 25 and 55 [10], and it affects an individual’s most economically productive years, posing a public health threat [11]. Migraine impacts approximately 10% of employed adults [12]. Evidence suggests that migraine significantly affects work performance and workplace relationships, leading to substantial productivity loss [10, 11, 13] and economic costs [14].

The annual economic costs arise from absenteeism, where migraine sufferers miss a full day of work due to migraine, and from reduced productivity (presenteeism), where individuals remain in the workplace but perform their duties at a lower level due to symptoms [15]. Both absenteeism and presenteeism significantly impair work-related ability, posing challenges for employees with migraine and their employers [11]. The chronic nature and prevalence of migraine are associated with increased functional impairment and a higher frequency of migraine attacks [16], which in turn correlate with greater productivity loss [10]. For instance, in the U.K., approximately 86 million workdays are lost annually due to migraine, resulting in an economic cost of £8.8 billion due to lost productivity [17]. Other European studies indicate that migraine sufferers experience, on average, around ten days of reduced productivity due to migraine attacks, missing approximately 3.5 workdays yearly, with an annual cost per migraine case of €1222, 93% of which (*i.e.*, €1136) is associated with absenteeism and limited productivity [18, 19].

Despite the significant impact of migraine on work-related activities, limited information exists regarding the specific types of activities primarily affected by migraine in the workplace [18]. A recent literature review indicated that skills such as problem-solving, speaking and driving, as well as remunerative employment are significantly affected by migraine [18].

To the best of our knowledge, few studies in Greece have investigated the burden of migraine on patients’ everyday lives, and none have focused on their professional lives, particularly in productivity-related domains [20, 21, 22, 23]. This gap is significant, given that over 0.6 million individuals in Greece experience migraine, resulting in considerable productivity loss. Only a small percentage seek care at specialized headache centers, with most being treated by non-experts [24]. A study in the Greek population also indicated that 58% of its participants reported severe disability [22].

Thus, this cross-sectional study aims to provide a better understanding of the overall impact of migraine among individuals in Greece, focusing on work performance and productivity. Accordingly, the objectives of this study are two-fold: (1) to explore the impact of migraine-related disability in everyday life in terms of missed days and productivity loss, and (2) to identify the work-related difficulties associated with migraine, as well as the potential factors that exacerbate these difficulties, providing a more detailed picture of the work-related challenges faced by individuals with migraine in Greek workplaces. By addressing this gap, this study will provide valuable insights that could raise awareness about social issues, emphasizing the impact of this health concern and informing better healthcare policy strategies and workplace interventions for migraine sufferers in Greece.

## 2. Materials and methods

### 2.1 Study population

The current cross-sectional questionnaire-based research was conducted between October 2023 and June 2024, involving a sample of 604 Greek outpatients suffering from migraine. Adult patients with migraine in Greece during the study period were recruited. The study population included adult employees from various workplaces, including education, social sciences, economic studies and medical sciences, as well as unemployed individuals with previous work experience. This diverse representation aimed to ensure that findings could be generalized across various professional and non-professional settings. The principal inclusion criterion for participant selection was a physician’s clinical migraine diagnosis of one primary migraine type (*e.g.*, migraine with aura or migraine without aura). Participants were only recruited for this study if they self-reported a previous migraine diagnosis confirmed by a neurologist. As part of the recruitment process, participants had to be medically diagnosed with migraine and complete a brief questionnaire based on the International Classification of Headache Disorders, 3rd Edition (ICHD-3) criteria. This questionnaire includes specific diagnostic criteria of migraine, such as frequency, duration, intensity and associated symptoms (*e.g.*, nausea, photophobia and phonophobia). Participants also had to verify the type of migraine they had been diagnosed with (*i.e.*, migraine with or without aura). Participants who did not meet these criteria were excluded from the study. Other inclusion criteria included being 18 years or older, being a resident of Greece, having access to the internet, and having present or past employment. Potential participants were excluded if they had secondary headaches or were unwilling to participate in the study. This approach aimed to comprehensively represent the population of adult patients with migraine in Greece, enhancing the potential generalizability of the findings. A total of 604 patients who met the inclusion criteria were recruited for this study.

### 2.2 Data collection instruments

The data collection process consisted of two phases. In the first phase, detailed socio-demographic data, such as sex, age, marital status, educational level and employment status, and clinical data, such as type of migraine, age of onset, frequency, duration and pain intensity, were systematically collected. The second phase involved administering two self-reported tools related to migraine. Specifically, research data regarding migraine were collected through the Migraine Disability Assessment (MIDAS) questionnaire [25, 26] and the 25-item version of the HEADWORK questionnaire [14, 27].

First, participants completed the MIDAS questionnaire, a simple, brief, self-administered tool developed by Stewart *et al*. [26] (1999) and Stewart *et al*. [25] (2000). Research has demonstrated its applicability in clinical practice [22, 26]. The MIDAS questionnaire is a seven-item questionnaire that captures information on migraine-related disability across different life domains over the previous three months. The first two items refer to the impact of headaches on work, in terms of missed workdays and days with decreased productivity by at least half. Notably, if productivity is decreased by 50% or more, the day is considered missed. The third and fourth items follow a similar scheme for household work. The fifth item concerns the number of days missed from leisure, family, or social activities due to headaches. The sixth item concerns the total number of headache days, and the seventh concerns average pain intensity. The MIDAS questionnaire is scored based on the sum of the days reported in the first five questions, with the resulting score classifying disability into four grades: Grade I, little or no disability (scores ranging from 0 to 5); Grade II, mild disability (scores ranging from 6 to 10); Grade III, moderate disability (scores ranging from 11 to 20); and Grade IV, severe disability (≥21). Two additional items of the MIDAS questionnaire (A and B) evaluate the frequency of headaches and pain intensity. These are not scored in the MIDAS questionnaire. The MIDAS questionnaire was translated to Greek and validated to provide a Greek version of the questionnaire for patients with migraine in Greece [22]. It was found to be valid and reliable, with good internal consistency [22]. The researchers in the current study chose this questionnaire because it is the most widely used outcome measure in headache research, offering valuable information on the number of headache days and average pain severity.

The HEADWORK questionnaire is the first instrument specifically designed to measure work-related disability in individuals with migraine [27]. The questionnaire is a 17-item, two-scale tool [14, 27]. It is divided into two sections. The first section, “Work-related difficulties”, includes 11 items addressing the impact of headaches on various work tasks and activities, including specific tasks such as using the computer and interacting with others, as well as general skills such as problem-solving. Patients are invited to respond on a five-point scale, ranging from 1 (no difficulty) to 5 (I cannot do it). The second section, “Factors contributing to work difficulties”, includes 6 items referring to personal, environmental and drug-related factors that may impair patients’ ability to perform their tasks. Respondents could also answer on a five-point scale, ranging from 1 (no limitations) to 5 (complete limitation). There is also an option “not applicable”, provided in cases where an activity or factor is irrelevant to a patient’s job. The HEADWORK questionnaire is a valid and reliable instrument that assesses the amount and severity of job-related difficulties and their associated factors. It is suitable for daily clinical practice, epidemiological research and clinical trials [27].

A pilot test (N = 10) was performed to assess the clarity and validity of the questionnaires and estimate the time needed for completion. Participants were asked to complete the MIDAS and HEADWORK questionnaires, with an average administration time of approximately 15 minutes. The researchers then asked questions regarding the clarity of the questions and whether there were difficulties encountered during completion, whether the time required was sufficient, and whether they had any suggestions for improvement. All participants provided positive feedback and did not indicate any significant need for improvement. Consequently, this pilot testing confirmed the reliability and comprehensibility of the selected questionnaires, established that their completion requires approximately 15 minutes, and allowed researchers to refine specific aspects relevant to the research aims, such as optimizing the wording of certain questions and enhancing the format for ease of use.

### 2.3 Data collection

The principles of the Helsinki Declaration of Biomedical Ethics guided this research initiative. Furthermore, formal approval for this research was obtained from the Ethical Committee of the University of Macedonia in Greece before the study commenced (Ref no: 6/24-11-2021). This research is an autonomous part of a broader investigation into the impact of migraine on work and the labor rights of migraine sufferers. Adherence to ethical standards ensures the integrity and reliability of the study’s findings.

Participants were comprehensively informed about the procedure and objectives of the study, both verbally and in written form, by the researchers. The researchers ensured that participation was voluntary, assuring the participants of their anonymity and the confidentiality of the gathered data. Participants were informed that they could withdraw from the study at any time and that all data would be kept private. All responses were collected and analyzed without identifiers. If participants had questions regarding the study, they were free to reach out through the contact information provided on the first page of the survey. The digital platform, Google Forms, was used for disseminating the questionnaires. Data collected through Google Forms were stored securely, with access restricted to the primary research team, ensuring confidentiality and data privacy. Notably, participants had the opportunity to review and change their answers before final submission via Google Forms. This feature enhanced the quality and accuracy of the collected data.

The convenience sampling method was employed to recruit study participants, with the researchers focusing on a specific population of adult patients with migraine that they could approach. The study participants were recruited through various channels. The Greek Society of Migraine and Headache Patients (GSMHP), a non-profit organization and a member of Pain Alliance Europe [28], assisted in implementing this research by sending out the Google Form link to all its members via their registered email address and encouraging broader outreach to the Greek population with migraine. Two reminders were sent to reinforce participation. The researchers also electronically distributed the questionnaires’ Google Form link via social media platforms, inviting individuals in Greece who met the inclusion criteria and were interested in the research to participate. In total, 604 recent and former employees participated in the study, with three incomplete and two inconsistent questionnaires excluded from the analysis. This approach aimed to achieve a representative sample of Greek patients with migraine, enhancing the generalizability of the findings.

### 2.4 Statistical analysis

This study assessed migraine-related disability across different life domains and work-related difficulties by analyzing the collected data. All data were anonymized before analysis. The data were coded and entered using Microsoft Office Excel 2010 (Microsoft Corporation, Redmond, WA, USA). All statistical analyses were performed using the IBM Statistical Package for the Social Sciences (SPSS) for Windows, version 20.0 (IBM Corp., Armonk, NY, USA). In this study, the authors employed simple descriptive statistical tests to describe the numerical variables of the sample as well as the frequency and percentage of non-numerical values. Mean and standard deviation (SD) are reported for continuous variables, while categorical variables are presented as absolute and relative frequencies. For continuous variables, summary statistics are tabulated. Chi-square tests and participant *t*-tests were performed to determine the differences in demographic characteristics and migraine disability. A *p*-value of < 0.05 was considered statistically significant. The normality of the distribution of continuous variables was examined using the Shapiro-Wilk test. The association of basic characteristics with the MIDAS total score and HEADWORK scales was evaluated using non-parametric statistical analysis with the Mann-Whitney U test. The correlation between MIDAS scores and HEADWORK scales was examined using Spearman’s correlation. While multiple comparisons were made, no correction (*e.g.*, Bonferroni) was done in this study since the analysis was exploratory and focused on identifying potential factors associated with work-related difficulties among individuals with migraine. Since this is a hypothesis-generating analysis, we decided not to apply stringent correction procedures, as they could potentially obscure meaningful associations. Future research may confirm these findings using stricter thresholds and correction procedures as needed.

Regarding Aim 2 of the study, the associations tested (*e.g.*, sex, marital status, employment, migraine type and episodic/chronic migraine) were selected to identify potential demographic and clinical factors that may contribute to work-related difficulties. This aligns with Aim 2, as it seeks to provide insight into individual differences in the challenges faced by migraine sufferers in Greek workplaces. By examining these aspects, the study provides important preliminary insights into the experiences of this population, establishing a framework for future research.

## 3. Results

Tables 1 and 2 detail the main socio-demographic and clinical characteristics of the sample. There were more female participants (533, 88.2%) than males (71, 11.8%), with a median (interquartile range, IQR) age of 42.0 (36.0, 48.0) years. Of those who responded to the relevant questions, 361 (59.8%) were married, 189 (31.3%) were unmarried and 46 (7.6%) were divorced. Regarding educational level, most of the participants (258, 42.7%) hold a master’s degree, while 219 (36.3%) have a university or technical degree. The vast majority of respondents (563, 93.2%) were gainfully employed, with 291 (48.2%) working in public service and 185 (30.6%) working in the private sector.

**Table 1.** Sociodemographic characteristics of the overall population.

		Total sample (N = 604)
Age (yr), median (IQR)		42.0 (36.0–48.0)
Age (yr), range		20.0–75.0
Sex, n (%)		
	Female	533 (88.2)
	Male	71 (11.8)
Marital status, n (%)		
	Unmarried	189 (31.3)
	Married	361 (59.8)
	Divorced	46 (7.6)
	Widow(er)	8 (1.3)
Educational level, n (%)		
	Secondary education/Lyceum (10–12 years of education)	85 (14.1)
	University/Technical college/College degree	219 (36.3)
	Master’s degree	258 (42.7)
	Doctor of philosophy (PhD)	42 (7.0)
Employment status, n (%)		
	Unemployed with previous work experience	41 (6.8)
	Employed	563 (93.2)
	-Public servant	291 (48.2)
	-Paid employee	185 (30.6)
	-Self-employed	87 (14.4)

*IQR: Interquartile Range.*

**Table 2.** Clinical characteristics of the overall population.

		Total sample (N = 604)
Can your migraine be described as migraine with or without aura? n (%)		
	Migraine without aura	337 (55.8)
	Migraine with aura	267 (44.2)
Can your migraine be described as chronic or episodic? n (%)		
	Episodic migraine	459 (76.0)
	Migraine without aura	269 (44.5)
	Migraine with aura	190 (31.5)
	Chronic migraine	145 (24.0)
	Migraine without aura	68 (11.3)
	Migraine with aura	77 (12.7)
Frequency of migraine, n (%)		
	Less than 1 episode per week	280 (46.4)
	1 episode per week	125 (20.7)
	More than 1 episode per week	165 (27.3)
	Daily	34 (5.6)
Duration of migraine without medication, n (%)		
	<3 h	75 (12.4)
	3–24 h	200 (33.1)
	1–2 d	162 (26.8)
	3 d	103 (17.1)
	>3 d	64 (10.6)
Intensity of migraine, n (%)		
	Mild	57 (9.4)
	Moderate	189 (31.3)
	Severe	271 (44.9)
	Very severe	87 (14.4)
Frequency of moderate/severe migraine pain, n (%)		
	Never	5 (0.8)
	Rarely	70 (11.6)
	Less than half the time	105 (17.4)
	About half the time or more than half the time	424 (70.2)
Nausea, n (%)		
	Never	84 (13.9)
	Rarely	188 (31.1)
	Less than half the time	126 (20.9)
	About half the time or more than half the time	206 (34.1)
Photophobia (sensitivity to light), n (%)		
	Never	55 (9.1)
	Rarely	120 (19.9)
	Less than half the time	123 (20.4)
	About half the time or more than half the time	306 (50.7)
Phonophobia (fear or sensitivity to sounds), n (%)		
	Never	69 (11.4)
	Rarely	117 (19.4)
	Less than half the time	134 (22.2)
	About half the time or more than half the time	284 (47.0)

Among the respondents, 337 (55.8%) experienced migraine without aura, while 267 (44.2%) reported migraine with aura. There was a predominance of episodic migraine, with 459 (76%) of the participants having episodic migraine and 24.0% having chronic migraine. Regarding migraine frequency, nearly half of the participants (280, 46.4%) experienced migraine attacks less than once a week; the duration of migraine varied without medication, with 33.1% reporting migraine lasting between 3 and 24 hours. Migraine intensity was notably severe, with 271 (44.9%) of migraineurs describing their migraine as severe and 87 (14.4%) describing it as very severe. Accompanying symptoms included moderate or severe pain, nausea, and sensitivity to light and sound.

MIDAS questionnaire scores are presented in Table 3 and provide valuable insights into the extent and impact of migraine-related disability among the participants. MIDAS Q1 addresses missed days of work or educational activities due to migraine. Most of the participants reported infrequent absences, with a low average score of 1.8 days (SD = 4.0), indicating that majority of them rarely missed work or educational activities. In contrast, MIDAS Q2 reported a higher level of reduced productivity at work or in educational settings, averaging approximately nine days (SD = 11.9). MIDAS Q3 focused on missed household work, while MIDAS Q4 measured days with reduced productivity in household tasks, showing similar results, with means of 8.5 (SD = 11.5) and 9.5 (SD = 12.5) days, respectively. MIDAS Q5 evaluated missed days of family, social or leisure activities due to headaches. According to the research data, participants missed approximately seven days (mean = 6.9, SD = 11.8) of familial and social activities. The median (IQR) MIDAS total score was 21.0 (10.0, 47.0), indicating high levels of migraine-related disability among this study’s participants. As shown in Table 4, majority of the participants had severe disability (52.2% for Grade IV). Findings regarding the frequency of headaches over the past three months (MIDAS A) revealed that most participants characterized their migraine as recurrent. MIDAS B assessed pain severity on a scale from 1 to 10, with most participants generally rating their migraine pain as severe. According to these results, the effect of migraine on daily activities across personal, household and professional levels, particularly in productivity-related domains, is substantial across the sample.

**Table 3.** MIDAS questionnaire scores of the overall population.

	Mean ± SD	Median (IQR)	Range
MIDAS 1: Number of days missed work	1.8 ± 4.0	0.0 (0.0–2.0)	0.0–45.0
MIDAS 2: Number of days with reduced work productivity	9.0 ± 11.9	5.0 (2.0–10.0)	0.0–90.0
MIDAS 3: Number of days missed household work	8.5 ± 11.5	5.0 (2.0–10.0)	0.0–88.0
MIDAS 4: Number of days with reduced productivity in household work	9.5 ± 12.5	5.0 (2.0–10.0)	0.0–90.0
MIDAS 5: Number of days missed social activities	6.9 ± 11.8	3.0 (1.0–7.0)	0.0–90.0
MIDAS total score (Total number of days MIDAS 1–5)	35.6 ± 40.9	21.0 (10.0–47.0)	0.0–347.0
MIDAS A: Number of headache days	16.1 ± 17.3	10.0 (5.0–20.0)	0.0–90.0
MIDAS B: Headache intensity	7.0 ± 1.8	7.0 (6.0–8.0)	1.0–10.0

*IQR: Interquartile Range; SD: standard deviation; MIDAS: Migraine Disability Assessment.*

**Table 4.** MIDAS disability grade of the overall population.

		Total sample (N = 604)
MIDAS disability grade, n (%)		
	Grade I (Little or no disability)	78 (12.9)
	Grade II (Mild disability)	78 (12.9)
	Grade III (Moderate disability)	133 (22.0)
	Grade IV (Severe disability)	314 (52.2)

*MIDAS: Migraine Disability Assessment.*

Table 5 reports the distribution of HEADWORK questionnaire items’ scores in the two sections. The median of the items’ scores was, for most single items, balanced between 2.0 and 4.0. Two scores were calculated based on the sum of the responses given to items in the two sections. The first score for Section A (*i.e.*, HEADWORK Score A), which refers to difficulties with work-related tasks, ranged from 13.0 to 65.0 (Median (IQR): 41.0 (31.0, 49.0)), while the second score for Section B (*i.e.*, HEADWORK Score B), which refers to factors hampering respondents’ workability, ranged from 12.0 to 58.0 (36.0 (28.0, 44.0)). Analyzing the difficulties due to headaches revealed that the most frequently reported issues were related to moderate difficulty in performing tasks such as talking and interacting with others (35.4%), understanding spoken information (33.4%), handling work problems (32.9%), and paying attention to work tasks (32.1%).

**Table 5.** HEADWORK questionnaire items score distribution of the overall population.

		Mean ± SD	Median (IQR)	Range
Item				
Difficulties due to headache		39.9 ± 12.5	41.0 (31.0–49.0)	13.0–65.0
	A1. Talking and interacting with other people	3.1 ± 1.1	3.0 (2.0–4.0)	1.0–5.0
	A2. Answering the phone	3.1 ± 1.2	3.0 (2.0–4.0)	1.0–5.0
	A3. Understanding what is said	2.6 ± 1.1	3.0 (2.0–3.0)	1.0–5.0
	A4. Dealing with work problems	3.1 ± 1.1	3.0 (2.0–4.0)	1.0–5.0
	A5. Reading and writing	2.8 ± 1.2	3.0 (2.0–4.0)	1.0–5.0
	A6. Using the PC	3.3 ± 1.2	3.0 (2.0–4.0)	1.0–5.0
	A7. Paying attention to work tasks	3.0 ± 1.1	3.0 (2.0–4.0)	1.0–5.0
	A8. Starting a new work task	3.3 ± 1.2	3.0 (2.0–4.0)	1.0–5.0
	A9. Solving organizational problems at work	3.2 ± 1.2	3.0 (2.0–4.0)	1.0–5.0
	A10. Moving from one place to another	3.1 ± 1.2	3.0 (2.0–4.0)	1.0–5.0
	A11. Driving a car	3.2 ± 1.3	3.0 (2.0–4.0)	1.0–5.0
Factors that prevented or limited work ability		35.3 ± 10.3	36.0 (28.0–44.0)	12.0–58.0
	B1. Air conditioning	2.6 ± 1.4	2.0 (1.0–3.0)	1.0–5.0
	B2. Negative attitudes of colleagues	3.0 ± 1.4	3.0 (2.0–4.0)	1.0–5.0
	B3. Brightness in the workplace	3.2 ± 1.3	3.0 (2.0–4.0)	1.0–5.0
	B4. Smell in the workplace	3.2 ± 1.3	3.0 (2.0–4.0)	1.0–5.0
	B5. Extended working hours	3.1 ± 1.4	3.0 (2.0–4.0)	1.0–5.0
	B6. Noise in the workplace	3.6 ± 1.2	4.0 (3.0–5.0)	1.0–5.0

*IQR: Interquartile Range; SD: standard deviation; PC: personal computer.*

Additionally, a notable number of individuals reported difficulty in solving organizational problems at work (29.3%) and using the computer (28.1%). A significant percentage of respondents also indicated that they could not perform certain activities, particularly driving a car (21.5%) and starting new work tasks (20.9%).

Several factors appear to significantly limit or prevent workability. Among environmental factors, air conditioning was found to cause no restriction for 28.8% of individuals, though 14.7% reported it as a significant limitation. Similarly, brightness and smell in the workplace caused substantial limitations for over 22% of employees. Noise was also a significant environmental challenge, with 25.8% reporting it as a significant limitation.

In contrast, work environment-related and personal factors presented different challenges. Negative colleague attitudes posed a considerable challenge, with 21% of participants reporting substantial limitations. Extended working hours had a lesser impact, with 19.5% of the participants, reporting no limitation. Overall, noise in the workplace emerged as significant limitation to workability, with 25.8% of individuals reporting it as substantial challenge.

Table 6 reports the results of the Mann-Whitney U test assessing differences in MIDAS total score and HEADWORK scales between various groups. As shown in Table 6, the MIDAS total score was significantly higher among females, those experiencing migraine with aura, and those with chronic migraine compared to those with episodic migraine. Similarly, HEADWORK Score A was higher among females and participants having migraine with aura, while HEADWORK Score B was higher among females, those having migraine with aura, and those with chronic migraine. However, no statistically significant differences in HEADWORK scores were detected based on marital or employment status. In contrast, a statistically significant difference in HEADWORK Score B was observed between episodic and chronic migraine sufferers (*p* < 0.001).

**Table 6.** Differences in MIDAS total score and HEADWORK scales based on selected variables (N = 604).

		MIDAS total score	*p*-value	HEADWORK score A: Difficulties due to headache	*p*-value	HEADWORK score B: Factors that prevented or limited work ability	*p*-value
Sex, median (IQR)							
	Females (Ν = 533)	24.5 (12.0–50.0)	<0.001*	41.0 (31.0–50.0)	0.013**	37.0 (29.0–44.0)	<0.001*
	Males (Ν = 71)	11.0 (5.0–27.0)		38.0 (28.0–45.0)		29.0 (21.0–40.0)	
Marital status, median (IQR)							
	Married (Ν = 361)	19.0 (9.0–41.0)	0.041**	40.0 (30.0–49.0)	0.412	35.0 (27.0–43.0)	0.326
	Unmarried/Divorced/Widow(er) (Ν = 243)	28.0 (12.0–51.0)		41.0 (32.0–50.0)		36.0 (29.0–44.0)	
Employment status, median (IQR)							
	Employed (Ν = 563)	21.0 (10.0–45.0)	0.202	41.0 (31.0–49.0)	0.653	36.0 (28.0–43.0)	0.258
	Unemployed (Ν = 41)	31.0 (10.0–55.0)		39.0 (30.0–49.0)		38.0 (27.0–48.0)	
Migraine type, median (IQR)							
	Migraine without aura (Ν = 337)	19.0 (9.0–41.5)	0.038**	39.0 (29.0–46.0)	<0.001*	34.0 (26.0–43.0)	<0.001*
	Migraine with aura (Ν = 267)	27.0 (13.0–50.0)		43.0 (35.0–52.0)		37.0 (30.0–45.0)	
Episodic/chronic migraine, median (IQR)							
	Episodic migraine (Ν = 459)	18.0 (9.0–38.0)	<0.001*	40.0 (30.0–49.0)	0.196	35.0 (26.0–42.0)	<0.001*
	Chronic migraine (Ν = 145)	43.0 (21.0–70.0)		42.0 (32.0–50.0)		41.0 (33.0–47.0)	

IQR: Interquartile Range; MIDAS: Migraine Disability Assessment. *Difference is significant at the 0.001 level (2-tailed); **Difference is significant at the 0.05 level (2-tailed); Mann-Whitney U test was used.

Table 7 reports separately the correlations between HEADWORK scales and MIDAS total score, headache frequency and average pain severity in the previous three months. All correlations were statistically significant, with HEADWORK scales being more strongly correlated with the MIDAS total score (*i.e.*, coefficients 0.20 with Score A and 0.40 with Score B; all *p* < 0.001) and average pain severity (*i.e.*, coefficients 0.33 with Score A and 0.38 with Score B; all *p* < 0.001) than with headache frequency (*i.e.*, coefficients 0.11 with Score A and 0.18 with Score B; all *p* < 0.05).

**Table 7.** Spearman correlation coefficients among test scores (N = 604).

Test scores	MIDAS total score	MIDAS A: Headache frequency	MIDAS B: Average pain severity	HEADWORK score A: Difficulties due to headache	HEADWORK score B: Factors that prevented or limited work
MIDAS total score	-	0.65*	0.27*	0.20*	0.40*
MIDAS A: Headache frequency	0.65*	-	0.14*	0.11**	0.18*
MIDAS B: Average pain severity	0.27*	0.14*	-	0.33*	0.38*
HEADWORK score A: Difficulties due to headache	0.20*	0.11**	0.33*	-	0.48*
HEADWORK score B: Factors that prevented or limited work	0.40*	0.18*	0.38*	0.48*	-

*Correlation is significant at the 0.001 level (2-tailed); **Correlation is significant at the 0.05 level (2-tailed). MIDAS: Migraine Disability Assessment.

## 4. Discussion

The current cross-sectional study explores the impact of migraine-related disability on everyday life in terms of missed days and productivity loss. It also identifies the extent of work-related difficulties experienced by individuals with migraine as well as the potential exacerbating factors. The research underscores the substantial impact of migraine on individuals’ personal, social and work lives.

The results indicate that migraine is a high-impact condition for most individuals. Disability assessment revealed that majority of the participants (52.2%) had severe disability (MIDAS Grade IV), while a significant proportion (22.0%) had moderate disability (MIDAS Grade III), highlighting the considerable prevalence of migraine-related disability. Several studies globally have confirmed these findings. Notably, a study showed that approximately one in four migraine sufferers may experience moderate to severe disability, with rates ranging from 12% in Europe to 19% in Asia, 22% in South America and 32.3% in North America [29]. Additionally, most participants reported severe and frequent headaches which exerted a significant effect on various aspects of their lives. As the frequency and severity of headache episodes increase, the number of days missed due to migraine also rises exponentially [11], along with the functional and social burden and productivity loss associated with migraine [30]. There is a tendency toward higher levels of reduced productivity with increased migraine severity [13].

Based on the MIDAS total score, the results reveal a significant burden of migraine among individuals in different areas of their everyday lives, including household responsibilities and social activities. It is evident that migraine directly impairs household functioning, with all the participants (N = 604) reporting being unable to perform housework for an average of 8.5 days (SD = 11.5) and experiencing decreased productivity for an average of 9.5 days (SD = 12.5) in the previous three months. This result aligns with that of a recent large-scale study which reported that 43.4% of its respondents were unable to complete housework on at least one day and 49.2% reported reduced productivity on at least one day [3]. The substantial impact of migraine on household tasks has also been reported by various other previous studies, indicating that individuals with migraine often struggle with daily household responsibilities [3, 31, 32]. Moreover, our findings revealed that participants missed approximately seven days of family, social, or leisure activities in the previous three months due to migraine. This finding is consistent with existing data showing that migraine exerts a substantial effect on social and familial life [3]. Furthermore, individuals with migraine frequently report that their condition negatively affects their roles as parents and partners, leading to missed family and social activities [33].

According to the findings of this study, higher MIDAS scores were strongly associated with greater work-related difficulties, as measured by the HEADWORK questionnaire. Participants with higher MIDAS scores faced significant challenges not only when they missed workdays but also when they experienced reduced productivity at work. The responses to the HEADWORK questionnaire indicated that individuals with greater migraine-related disability reported increased difficulties in completing their work tasks. Furthermore, these individuals often mentioned higher levels of presenteeism, characterized by decreased productivity. Existing research supports these findings, highlighting a strong correlation between MIDAS scores and work-related disability. According to a recent study, individuals with higher migraine-related disability encounter difficulties in performing their work duties, with absenteeism and decreased productivity being common concerns [14]. Moreover, another study assessing the impact of migraine on work productivity indicated that participants with higher MIDAS scores experienced significant work disability [11].

Our study suggests that the personal and social burdens of migraine may directly affect employees’ work-related performance. As participants in a relevant study noted, being unable to complete household tasks and experiencing reduced productivity at the family and social levels may extend into the workplace [34]. The missed days from family or social activities due to migraine could lead to increased stress and decreased ability to perform work-related duties effectively [3], contributing to reduced concentration and efficiency in the work environment [35]. It is well-established that migraine causes impaired functioning in work productivity and leads to occupational disability [11]. The reduced productivity likely impacts patients’ career choices, job status and/or stability, financial status, workplace relationships, mood and self-confidence [10].

Migraine results in a substantial number of lost workdays each year [11, 36]. However, most participants in our study avoided migraine-related absenteeism from work or educational settings. This finding aligns with previous studies that have reported migraine sufferers exhibiting higher levels of presenteeism compared to absenteeism [11, 13, 31]. When individuals with migraine experience symptoms, they often choose to continue working [18]. Employees frequently take analgesics to attend work, often at the expense of their productivity [13]. Our study corroborates this finding, as participants reported attending work or educational settings despite significant reductions in their productivity due to headaches, reinforcing the condition of presenteeism. This may be attributed to several factors. Many individuals with migraine may perceive stigma associated with their condition, fearing that it might be viewed as an excuse for missing work or social events [37]. Likewise, individuals with migraine may conceal their symptoms due to shame or a lack of confidence in society [37].

Overall, migraine adversely affects individuals’ ability to perform their professional duties effectively [38, 39]. Over 10% of the adult working population in Greece suffers from debilitating headaches that impact work productivity [21]. Currently employed individuals who suffer from migraine experience greater impairment in work productivity compared to those without migraine [31, 40]. Our findings suggest a high burden of migraine among employees, as work-related difficulties are associated with migraine attacks [41]. Most participants reported moderate to high levels of specific work-related difficulties due to migraine. According to the HEADWORK questionnaire results, migraine sufferers face several challenges related to cognitive and interpersonal functions that impact their ability to perform work tasks. Specifically, participants reported difficulties in talking, interacting with others and using computers. Additionally, answering the phone posed a significant challenge for over 40% of participants. One study, consistent with our findings, reported that migraine affects the ability of sufferers to communicate at work [38]. Simultaneously, evidence indicates that increased screen time on computers may trigger migraine episodes [38].

Some findings also emphasize severe limitations in complex cognitive functioning, such as starting new work tasks, solving organizational problems and addressing work-related issues. These findings align with the existing literature, which indicates that migraine is associated with impaired problem-solving abilities [38]; other evidence reports difficulties in cognitive functioning during work [39]. The literature suggests that skills such as problem-solving and activities such as speaking are significantly affected by migraine [18, 42]. According to previous research, 40% of participants reported difficulties with attention, executive function, processing speed, and memory on headache days, negatively impacting their work productivity [35, 43]. The ability to read and write also tends to be affected by migraine, as indicated by both previous studies [31] and this study. Driving is also substantially affected, with several participants reporting an inability to drive due to migraine. This finding is consistent with that of relevant studies that highlight the significant impact of migraine on driving [3, 18]. In a recent study, participants reported being unable to drive due to the unpredictability and intensity of their migraine episodes [38].

The current research further identifies environmental and interpersonal factors that impact work performance. It emphasizes that work-related factors are not only potential triggers of migraine attacks but also contextual elements that create a challenging environment for migraine sufferers to meet their job demands [39]. Previous studies have confirmed that environmental factors such as intense noise, bright light and strong odors can trigger migraine episodes [13, 44, 45], a similar observation from our study. Furthermore, negative attitudes from colleagues pose a substantial challenge that might influence the work performance of employees suffering from migraine. The Migraine in the Workplace Survey conducted by the Migraine Association of Ireland in 2021, reported that less than half of participants felt supported at work, and many colleagues perceived migraine as merely a headache [43]. Additionally, employees with migraine often face stigma from colleagues or employers [37, 38].

Regarding interpersonal factors, extended working hours emerged as significant limitation to work in the present study. This finding is corroborated by existing research, which has indicated that long working hours are associated with a higher risk of developing a migraine [38]. Among the top work-related stressors are violence at work, traffic accidents, injuries, trauma, excessive effort, poor rewards, intrusive leadership and off-time work [41]. Stress and anxiety are among the most common work limitations, often exacerbated by a lack of understanding from colleagues [38, 43]. Moreover, some employees with migraine have opted to reduce their working hours and work part-time due to their condition [10]. Night work is likely a risk factor for migraine [41]. Existing research indicates that migraine is more prevalent among night shift workers than day workers [46].

This study indicates that specific demographic and clinical characteristics influence the levels of disability experienced due to migraine. Specifically, regarding sex differences, the current study’s findings align with those of a prior research in Greece, which revealed that migraine is predominantly observed in women [21]. Notably, females present 1.69 times higher odds of being diagnosed with migraine [11]. This higher prevalence of migraine among women compared to men may be attributed to physiological and hormonal factors [47]. This finding underscores the need for a gender-specific approach to migraine risk prevention and care [11]. Migraine has a pronounced impact on female participants compared to male participants [32, 41]. In a recent analysis, women were found to be more likely to report an inability to perform tasks and participate in social or family activities [3]. The impact of headaches on productivity loss is also significantly higher in females than in males [41, 45]. Specifically, according to the MIDAS questionnaire used in the current study, females indicated greater levels of migraine-related disability than males. Overall, studies consistently support this finding [30, 48].

Marital status also impacted migraine-related disability, albeit to a lesser extent. In the current study, unmarried, divorced or widowed participants had higher MIDAS scores than married participants. A study found that as monthly headaches increased, the percentage of migraine sufferers who were married or employed decreased [49]. These findings suggest a possible relationship between migraine-related disability and social support. Social support can lead to a better understanding of migraine [44]. However, marital status did not significantly affect difficulties due to headaches or workplace limitations.

The research reported higher MIDAS total scores in individuals with chronic migraine, indicating reduced functioning. This finding aligns with that of a recent study, which found the impact of chronic migraine to be more significant than that of episodic migraine [18]. Participants with chronic migraine also reported more factors limiting their ability to work compared to those with episodic migraine. However, there was no significant difference between the two groups of migraine sufferers regarding work-related difficulties due to headaches. An earlier study reported that both episodic and chronic migraine have a substantial effect on daily and workplace activities [15]. According to a scoping review, both chronic and episodic migraine negatively affect work-related productivity [18, 50]. Notably, in the present study, there were no statistically significant differences in migraine-related disability with regard to employment status.

The present research has several limitations that should be considered. The study is cross-sectional, providing data at a single point in time, which restricts the ability to assess potential causality between migraine and work-related disability. Longitudinal studies are recommended for future research, as they would facilitate the evaluation of the relationship between migraine and work-related disability over time. Additionally, the participants in this study were recruited using a convenience sampling method, which may have introduced selection bias; the sample may not accurately represent the Greek population of migraine sufferers, particularly those without internet access. The researchers attempted to mitigate this bias and collect a representative sample through social media and the mediation of the Greek Society of Migraine and Headache Patients, an organization with members all over Greece. Moreover, this sampling method has been effectively utilized in several studies to gather substantial data [51]. Internet surveys have become the most common method for collecting qualitative data due to various advantages, including lower costs, faster implementation and greater efficiency in data analysis [52]. Furthermore, as the research focuses on the Greek population, cultural and socioeconomic differences should be considered as potential influential factors in the results. The current study’s sample exhibits unique characteristics that deviate from those in other studies. Specifically, a high proportion of participants (85%) hold university or higher degrees. This may reflect Greece’s status as one of the European countries with the highest number of tertiary education students and graduates, which could be reflected in the sample’s demographic characteristics [53]. Additionally, while nearly two-thirds of the participants reported experiencing fewer than four migraine days per month, over half reported a MIDAS Grade of IV. This discrepancy raises questions about the classification of disability levels concerning sporadic migraine episodes. Even infrequent migraines can cause significant disability, highlighting the diversity of migraine episodes and their impact on daily life [54]. Given that these findings diverge from those in the existing literature [54, 55], which provide strong evidence of the correlation between migraine frequency and MIDAS scores, further examination is warranted. Moreover, a significant proportion of the sample identified as migraineurs with aura (44%). However, it is important to note that self-reported diagnoses of migraine with aura, without appropriate clinical assessment, may lead to overreporting [56]. Further investigation is needed to comprehensively understand the needs and challenges faced by patients with migraine, particularly in workplace settings. Future studies could incorporate quantitative methods to gain a deeper understanding of the relationship between migraine characteristics and disability. Notably, substantial research already exists in this area [43, 57, 58], providing significant findings on how migraine-related disability affects functionality in workplace settings. Additionally, further research may emphasize existing measures and explore new approaches to identify patients’ specific work-related challenges. This may support the establishment of more targeted interventions and measures to assist individuals with migraine in the workplace.

The current study highlights the considerable impact of migraine on various aspects of life, particularly in workplace settings. The findings could serve as a starting point through which all stakeholders in Greece—policymakers, healthcare providers and patients with migraine and their families, employers and coworkers—recognize the issue and its effects and address it appropriately. The stigma surrounding migraine [37] underscores the magnitude of the problem, and its impact underscores the need for comprehensive strategies to mitigate its effects (including economic implications such as missed hours and productivity loss), not only for patients but also for society as a whole. Workplace accommodations, such as flexible schedules and working environments designed to minimize triggers like noise and brightness, are critical for supporting employees with migraine. Policymakers could utilize these findings to raise public awareness through informative campaigns on migraine and to incorporate legal protections that reduce economic losses from presenteeism and absenteeism. Healthcare providers should prioritize developing treatment plans that address not only the physical symptoms but also the psychosocial factors associated with migraine. There is an urgent need for developing and implementing educational workplace programs [35], which have been linked to reduced presenteeism [43]. The costs associated with productivity losses and high employer expenses highlight the importance of these programs in raising awareness, fostering understanding and reducing the stigma, burden and costs associated with lost workplace productivity due to migraine [35]. Employers should consider implementing educational programs to mitigate stigma and promote understanding and support for migraine sufferers in their workplaces. Migraine is not merely a peculiarity; it is not an excuse, or a headache, but a serious medical condition that may reduce attendance and decrease productivity. Our findings prove its impact and the importance of appropriate interventions.

## 5. Conclusions

This study underscores the substantial and multifaceted impact of migraine on individuals’ professional, personal, and social lives. Migraine is prevalent among adult employees, leading to significant productivity losses and impaired work performance. Although migraine may not cost individuals entire days of work or school, it still results in diminished effectiveness when it occurs. Utilizing instruments such as the MIDAS and HEADWORK questionnaires, this research has highlighted key areas where individuals with migraine face considerable challenges, particularly work-related productivity and social relationships. Workplace accommodations, increased migraine awareness and targeted interventions could help address these challenges. Future research should focus on longitudinal assessments to evaluate the impact of the recommended interventions and develop strategies to improve the quality of life of individuals with migraine.
